# The phosphatidylethanolamine derivative diDCP-LA-PE mimics intracellular insulin signaling

**DOI:** 10.1038/srep27267

**Published:** 2016-06-02

**Authors:** Tomoyuki Nishizaki, Akinobu Gotoh, Tadashi Shimizu, Akito Tanaka

**Affiliations:** 1 Innovative Bioinformation Research Organization, Kobe, Japan; 2 Laboratory of Cell and Gene Therapy, Institute for Advanced Medical Sciences, Hyogo College of Medicine, Nishinomiya, Japan; 3 Laboratory of Chemical Biology, Advanced Medicinal Research Center, Hyogo University of Health Sciences, Kobe, Japan

## Abstract

Insulin facilitates glucose uptake into cells by translocating the glucose transporter GLUT4 towards the cell surface through a pathway along an insulin receptor (IR)/IR substrate 1 (IRS-1)/phosphatidylinositol 3 kinase (PI3K)/3-phosphoinositide-dependent protein kinase-1 (PDK1)/Akt axis. The newly synthesized phosphatidylethanolamine derivative 1,2-*O*-bis-[8-{2-(2-pentyl-cyclopropylmethyl)-cyclopropyl}-octanoyl]-*sn*-glycero-3-phosphatidylethanolamine (diDCP-LA-PE) has the potential to inhibit protein tyrosine phosphatase 1B (PTP1B) and to directly activate PKCζ, an atypical isozyme, and PKCε, a novel isozyme. PTP1B inhibition enhanced insulin signaling cascades downstream IR/IRS-1 by preventing tyrosine dephosphorylation. PKCζ and PKCε directly activated Akt2 by phosphorylating at Thr309 and Ser474, respectively. diDCP-LA-PE increased cell surface localization of GLUT4 and stimulated glucose uptake into differentiated 3T3-L1 adipocytes, still with knocking-down IR or in the absence of insulin. Moreover, diDCP-LA-PE effectively reduced serum glucose levels in type 1 diabetes (DM) model mice. diDCP-LA-PE, thus, may enable type 1 DM therapy without insulin injection.

Insulin activates IR, a receptor tyrosine kinase (RTK), to phosphorylate its own receptor and IRS-1 at the tyrosine residue. Phosphorylated IRS-1 recruits and activates PI3K, which produces phosphatidylinositol (3,4,5)-triphosphate [PI(3,4,5)P_3_] by phosphorylating phosphatidylinositol 4,5-bisphosphate [PI(4,5)P_2_] and in turn, PI(3,4,5)P_3_ binds to and activates PDK1. PI3K and PDK1 activate Akt2 by phosphorylating at Ser474 and Thr309, respectively^1^. Akt plays a pivotal role in the regulation of GLUT4 delivery to the cell surface and glucose uptake into cells.

We have earlier found that the phosphatidylethanolamine derivative diDCP-LA-PE, with 8-[2-(2-pentyl-cyclopropylmethyl)-cyclopropyl]-octanoic acid (DCP-LA) on the α and β positions, serves as a PTP1B inhibitor and a PKC activator^2^. diDCP-LA-PE activated PKCα, -βI, -βII, -γ, -δ, -ε, -λ/ι, and -ζ in a concentration (1–100 μM)-dependent manner. Notably, activation of PKCλ/ι and -ζ was not obtained with other phospholipid derivatives 1,2-*O*-bis-DCP-LA-*sn*-glycero-3-phosphatidyl-L-serine (diDCP-LA-PS), 1,2-*O*-bis-DCP-LA-*sn*-glycero-3-phosphatidylcholine (diDCP-LA-PC) and 1,2-*O*-bis-DCP-LA-*sn*-glycero-3-phosphatidyl-D-1-inositol (diDCP-LA-PI). To our knowledge, direct activator of atypical PKC isozymes PKCλ/ι and -ζ has not been provided so far.

PTP1B inhibition would cause a persistent tyrosine phosphorylation of IR/IRS-1, thereby enhancing the ensuing downstream signaling. Indeed, diDCP-LA-PE enhanced IR phosphorylation at Tyr1185 and IRS-1 phosphorylation at Tyr1222, although the latter was not significant. PKC, on the other hand, is implicated in the regulation of GLUT4 trafficking through an insulin signaling pathway^3^. In response to insulin PKCλ/ι and -ζ, are activated and promote GLUT4 translocation towards the cell surface^4^,^5^,^6^,^7^,^8^. Insulin activates PKCα, -βII, and -δ as well, which regulate GLUT4 trafficking^9^,^10^. Moreover, PKCε may also participate in the regulation of GLUT4 trafficking^11^. We, in the light of these facts, postulated that diDCP-LA-PE might mimic intracellular insulin signaling.

The present study was conducted to prove this hypothesis. We show here that diDCP-LA-PE facilitates glucose uptake into differentiated 3T3-L1 adipocytes still with knocking-down IR or in the absence of insulin and reduces serum glucose levels in type 1 DM model mice without insulin injection.

## Materials and Methods

### Approval of experiments

All experimental protocols were approved by Hyogo College of Medicine. All procedures using animals have been approved by the Animal Care and Use Committee at Hyogo College of Medicine and were in compliance with the National Institutes of Health Guide for the Care and Use of Laboratory Animals.

### Cell culture

3T3-L1-GLUT4myc fibroblast cell line (kindly provided by Prof. Ebina at University of Tokushima, Japan), expressing GLUT4myc that is constructed by inserting a human c-MYC epitope (14 amino acids) into the first ectodomain of GLUT4. Cells were cultured by the method as described previously^1^. We have confirmed in the Oil-Red O staining and Western blot analysis using an anti-peroxisome proliferator-activated receptor γ antibody that cells used here are well differentiated into 3T3-L1 adipocytes.

### Western blotting

Western blotting was carried out in differentiated 3T3-L1-GLUT4myc adipocytes using antibodies against phospho-Thr308/309-Akt1/2 [pT308(9)] (Cell Signaling Technology, Inc., Danvers, MA, USA), phospho-Ser473/474-Akt1/2 [pS473(4)] (Cell Signaling Technology) and Akt1/2 (Cell Signaling Technology) by the method as previously described^1^.

### Monitoring of GLUT4 trafficking

GLUT4 trafficking in differentiated 3T3-L1-GLUT4myc adipocytes was monitored by the methods as described previously^1^. Differentiated 3T3-L1 adipocytes were incubated in Krebs-Ringer-HEPES buffer (136 mM NaCl, 4.7 mM KCl, 1.25 mM CaCl_2_, 1.25 mM MgSO_4_ and 20 mM HEPES, pH 7.5) containing 0.2% (w/v) bovine serum albumin supplemented with 10 mM glucose for 1 h at 37 °C. Cells were treated with insulin or a variety of lipids in the presence and absence of inhibitors for 20 min. Then, cells were homogenized by sonication in an ice-cold mitochondrial buffer [210 mM mannitol, 70 mM sucrose, and 1 mM EDTA, 10 mM HEPES, pH 7.5] containing 1% (v/v) protease inhibitor cocktail (Nacalai Tesque, Kyoto, Japan) and subsequently, homogenates were centrifuged at 3,000 rpm for 5 min at 4 °C. The supernatants were centrifuged at 11,000 rpm for 15 min at 4 °C and further, the collected supernatants were ultracentrifuged at 100,000 *g* for 60 min at 4 °C to separate the cytosolic and plasma membrane fractions. The supernatants and pellets were used as the cytosolic and plasma membrane fractions, respectively. Whether the cytosolic and plasma membrane components were successfully separated was confirmed in the Western blot analysis using antibodies against the cytosolic marker lactate dehydrogenase (LDH) and the plasma membrane marker cadherin. The cytosolic fraction contains GLUT4 in transport vesicles as well as in intracellular compartments such as the endosomes and the trans-Golgi network, and the plasma membrane fraction otherwise contains GLUT4 on the plasma membrane, but not in a partial pool near the plasma membrane.

Protein concentrations for each fraction were determined using a BCA protein assay kit (Thermo Fisher Scientific, Waltham, MA, USA). Proteins in the plasma membrane fraction were resuspended in the mitochondrial buffer containing 1% (w/v) sodium dodecyl sulfate (SDS). Proteins for each fraction were separated by SDS-polyacrylamide gel electrophoresis (SDS-PAGE) and transferred to polyvinylidene difluoride (PVDF) membranes. After blocking with TBS-T [150 mM NaCl, 0.1% (v/v) Tween-20, and 20 mM Tris, pH 7.5] containing 5% (w/v) bovine serum albumin (BSA), blotting membranes were reacted with an anti-c-myc antibody (Merck Millipore, Darmstadt, Germany) followed by a horseradish peroxidase (HRP)-conjugated goat anti-mouse IgG antibody. Immunoreactivity was detected with an ECL kit (Invitrogen, Carlsbad, CA, USA) and visualized using a chemiluminescence detection system (GE Healthcare, Piscataway, NJ, USA). Signal density was measured with an ImageQuant software (GE Healthcare).

### Construction and transfection of siRNA

The siRNAs to silence each targeted genes for IR (5′CCUACACUUUHCUAAUCAtt-3′ and 5′-UGAUUGAGCAAAGUGUAGGcc-3′), PI3K p85α (PI3K) (5′-GCGAAUGAUAUGUAUCAGAtt-3′ and 5′-UCUGAUACAUAUCAUUCGCtc-3′), PDK1 (5′-CCUCGUUUAUGUUUCUGCGtt-3′ and 5′-CGCAGAAACAUAAACGAGGtc-3′), Akt1/2 (siRNA sequence: not provided), PKCλ/ι (siRNA sequence: not provided), PKCζ (5′-GGACCUCUGUGAGGAAGUGtt-3′ and 5′-CACUUCCUCACAGAGGUCCtt-3′), PKCε (5′-GCACUUGCGUUGUCCACAAtt-3′ and 5′-UUGUGGACAACGCAAGUGCaa-3′), PKCγ (5′-ACAAGUUACUGAACCAGGAtt-3′ and 5′-UCCUGGUUCAGUAACUUGUac-3′) and mTOR (5′-GAAUGGUGUCGAAAGUACAtt-3′ and 5′-UGUACUUUCGACACCAUUCtt-3′) were obtained from Santa Cruz Biotechnology (Santa Cruz, CA, USA) and the negative control (NC) siRNA, which has the scrambled sequence with the GC content and nucleic acid composition same as those for siRNA for each protein, was from Ambion (Carlsbad, CA, USA). siRNAs were transfected into differentiated 3T3-L1-GLUT4myc adipocytes using a Lipofectamine reagent, and cells were used for experiments 48 h after transfection.

### Cell-free kinase assay

PKC activity in the cell-free systems was quantified by the method as previously described^2^,^12^. Briefly, synthetic PKC substrate peptide (Pyr-Lys-Arg-Pro-Ser-Gln-Arg-Ser-Lys-Tyr-Leu; MW, 1,374) (Peptide Institute Inc., Osaka, Japan) (10 μM) was reacted with human recombinant PKCα, -βI, -βII, -γ, -δ, -ε, -λ/ι or -ζ in a medium containing 20 mM Tris-HCl (pH 7.5), 5 mM Mg-acetate, 10 μM ATP, and diDCP-LA-PE in the absence of phosphatidylserine and diacylglycerol at 30 °C for 5 min. Activity for novel PKCs such as PKCδ and -ε was assayed in Ca^2+^-free medium and activity for the other PKC isozymes in the medium containing 100 μM CaCl_2_. After loading on a reversed phase high performance liquid chromatography (LC-10ATvp; Shimadzu Co., Kyoto, Japan), a substrate peptide peak and a new product peak were detected at an absorbance of 214 nm. Areas for non-phosphorylated and phosphorylated PKC substrate peptide were measured (total area corresponds to concentration of PKC substrate peptide used here), and the amount of phosphorylated substrate peptide was calculated. The amount of phosphorylated substrate peptide (pmol/1 min) was used as an index of PKC activity.

In the cell-free Akt2 assay, human recombinant Akt2 (Active Motif, Carlsbad, CA, USA) was reacted diDCP-LA-PE in a medium containing 25 mM 3-morpholinopropanesulfonic acid (pH 7.2), 25 mM MgCl_2_, 12.5 mM glycerol 2-phosphate, 5 mM EGTA, 2 mM EDTA, 0.25 mM dithiothreitol, and 250 μM ATP containing PKCγ, -λ/ι, -ζ or -ε at 30 °C for 20 min. Phosphorylated Akt1/2 was quantified by Western blotting using antibodies against pT308(9) (Cell Signaling Technology), pS473(4) (Cell Signaling Technology), and Akt1/2 (Cell Signaling Technology).

### Glucose uptake assay

Glucose uptake assay was carried out by the method as described previously^1^,^13^,^14^. Differentiated 3T3-L1-GLUT4myc adipocytes without and with IR knock-down were incubated in a Krebs-Ringer-HEPES buffer containing 0.2% (w/v) BSA supplemented with 10 mM glucose at 37 °C for 1 h. Then, cells were not treated and treated with diDCP-LA-PE or insulin in phosphate-buffered saline supplemented with 10 mM glucose at 37 °C for 2 h. After treatment, extracellular solution was collected and glucose was labeled with *p*-aminobenzoic ethyl ester (ABEE), followed by HPLC. Glucose concentration taken up into cells was calculated by subtracting extracellular glucose concentration from initial extracellular glucose concentration (10 mM).

### Oral glucose tolerance test (OGTT)

Streptozotocin, which exerts its cytotoxic effect on pancreatic β cells, is a chemical inducer of experimental DM in rodents. C57BL/6J mice (male, 8 weeks of age) (Japan SLC Inc., Shizuoka, Japan) were intraperitoneally injected with a single streptozotocin (250 mg/kg) and used as a type 1 DM model mice 4 days after injection. For normal control group, mice were injected with saline.

C57BL/KsJ-leprdb/leprdb mice are a well-established genetic model of type 2 DM, which have characteristics similar to human type 2 DM including obesity, hyperglycemia, and extreme insulin resistance. The mice are obese and hyperinsulinemic up to 1 month of age, then insulin resistance worsens with the appearance of hyperglycemia. C57BL/KsJ-leprdb/leprdb and wild-type C57BL/6J mice (female, 8 weeks) were purchased from CLEA Japan (Tokyo, Japan) and used as a type 2 DM model mice and normal control mice, respectively.

In OGTT, mice were fasted for 12 h, followed by oral administration with diDCP-LA-PE using a feeding needle or intraperitoneal injection with insulin 30 min prior to loading glucose. After collection of blood (10 μL) from the tail vein, the serum was labeled with ABEE and loaded onto the HPLC system and glucose concentrations were calculated from the peak area/concentration calibration curve made before using a standard glucose solution.

### Statistical analysis

Statistical analysis was carried out using unpaired *t*-test, analysis of variance (ANOVA) followed by a Bonferroni correction and ANOVA followed by Fisher’s protected least significant difference (PLSD) test.

## Results

### diDCP-LA-PE activates Akt1/2 in differentiated 3T3-L1 adipocytes

Akt, a serine/threonine protein kinase, includes three members Akt1, -2 and -3, and of them Akt2 is abundantly expressed in adipocytes and skeletal muscle cells and regulates GLUT4-mediated glucose uptake^15^,^16^. Akt1, -2 and -3 are activated by being phosphorylated at Thr308, -309 and -305 and Ser473, -474 and -472, respectively.

**Figure 1:**
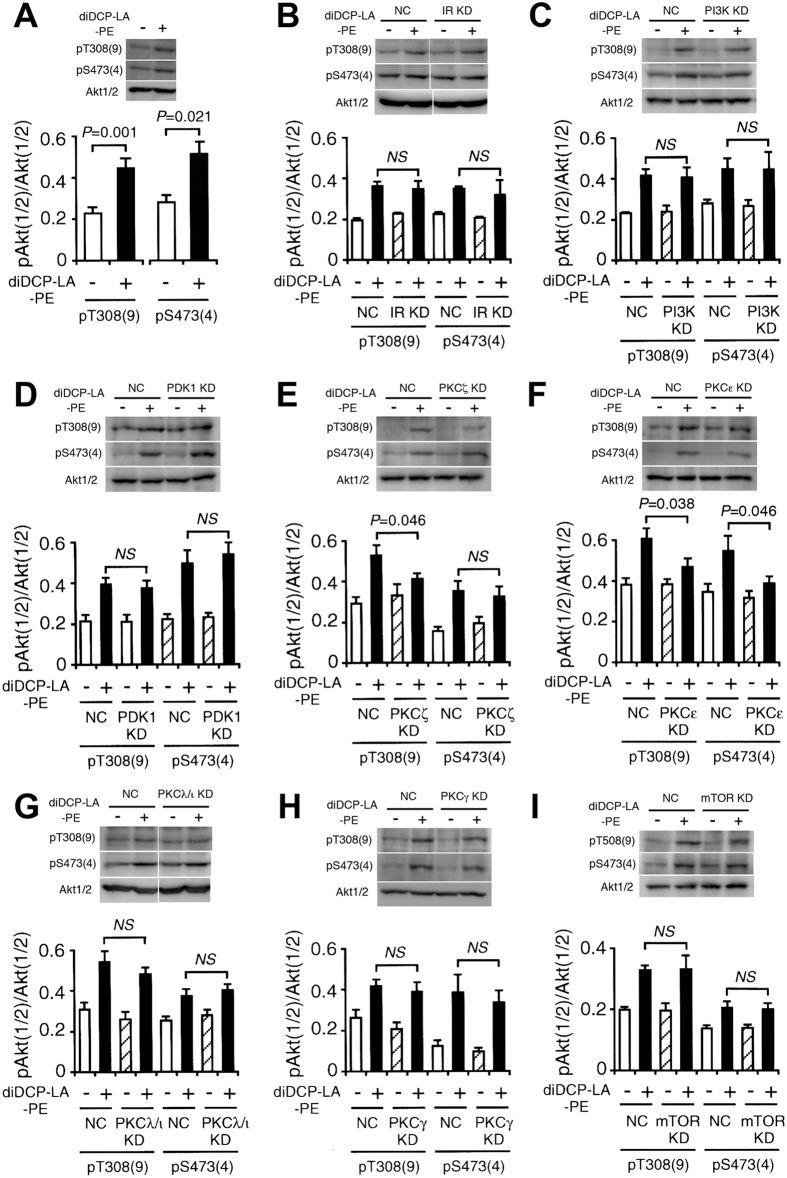
diDCP-LA-PE activates Akt1/2 in a PKCζ- or PKCε-dependent manner. Differentiated 3T3-L1-GLUT4myc adipocytes, which were non-transfected (**A**) and transfected with siRNAs for IR (**B**), PI3K (**C**), PDK1 (**D**), PKCζ (**E**), PKCε (**F**), PKCλ/ι (**G**), PKCγ (**H**) or mTOR (**I**) were treated with diDCP-LA-PE (1 μM) for 10 min followed by Western blotting. In the graphs, each column represents the mean (±SEM) signal intensity for phosphorylation at Thr308/309 [pT308(9)] or Ser473/474 [pS473(4)] relative to that for Akt1/2 (n = 4–6 independent experiments). *P* values, ANOVA followed by a Bonferroni correction. *NS*, not significant. NC, negative control; KD, knock-down.

**Figure 2:**
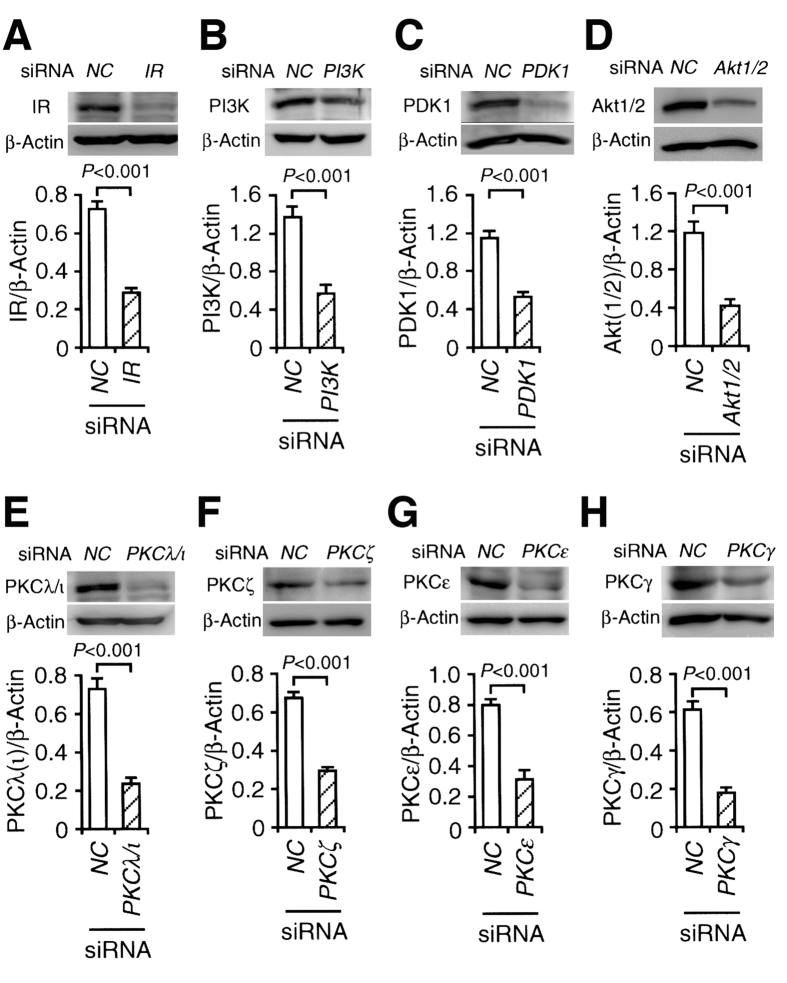
siRNA knocking-down efficacy. Differentiated 3T3-L1-GLUT4myc adipocytes were transfected with siRNAs for negative control (NC) and IR (**A**), PI3K (**B**), PDK1 (**C**), Akt1/2 (**D**), PKCλ/ι (**E**), PKCζ (**F**), PKCε (**G**) or PKCγ (**H**), and 48 h later Western blotting was carried out. The signal intensity for each protein was normalized by that for β-actin. In the graphs, each column represents the mean (±SEM) normalized intensity (n = 4 independent experiments). *P* values, unpaired *t*-test.

diDCP-LA-PE significantly enhanced phosphorylation of Akt1/2 both at Thr308/309 and Ser473/474 in adipocytes differentiated from 3T3-L1-GLUT4myc fibroblasts (Fig. 1A), confirming diDCP-LA-PE-induced activation of Akt1/2. To understand the mechanism underlying diDCP-LA-PE-induced Akt1/2 activation, we knocked-down a variety of the related proteins. In knock-down experiments, we have confirmed that expression of the target proteins are actually suppressed in cells transfected with each siRNA (Fig. 2). diDCP-LA-PE-induced phosphorylation of Akt1/2 at Thr308/309 and Ser473/474 was not affected by knocking-down IR (Fig. 1B). This implies that diDCP-LA-PE is capable of activating Akt1/2, regardless of IR. Unexpectedly, diDCP-LA-PE-induced Akt1/2 phosphorylation was not inhibited by knocking-down PI3K and PDK1 (Fig. 1C,D). This suggests additional pathways independent of PTP1B inhibition for diDCP-LA-PE-induced Akt activation. In contrast, the Thr308/309 phosphorylation was significantly suppressed by knocking-down PKCζ, but not another atypical PKC isozyme PKCλ/ι (Fig. 1E,G). Surprisingly, phosphorylation both at Thr308/309 and Ser473/474 was clearly suppressed by knocking-down PKCε, a novel PKC isozyme (Fig. 1F). These results indicate that PKCζ and PKCε participate in the activation of Akt1/2. diDCP-LA-PE-induced Akt1/2 phosphorylation was not affected by knocking-down PKCγ, a conventional PKC isozyme (Fig. 1H). This supports the notion that Akt1/2 activation is mediated by PKCζ and PKCε.

Mammalian target of rapamycin complex 2 (mTORC2), which is activated by PI3K, is shown to phosphorylate Akt1/2 at Ser473/474^17^,^18^,^19^. diDCP-LA-PE-induced Akt1/2 phosphorylation at Ser473/474 was not attenuated by knocking-down mTOR (Fig. 1I), which rules out the implication of mTOR in diDCP-LA-PE-induced Akt1/2 activation. IKBKE and Pak1 are also recognized to phosphorylate Akt1/2 at Ser473/474 in a PI3K-independent manner^20^,^21^. It, however, is presently unknown whether IKBKE and Pak1 participate in the diDCP-LA-PE-induced serine phosphorylation of Akt1/2.

### Cooperation of PKCζ and PKCε directly and fully activates Akt2

**Figure 3:**
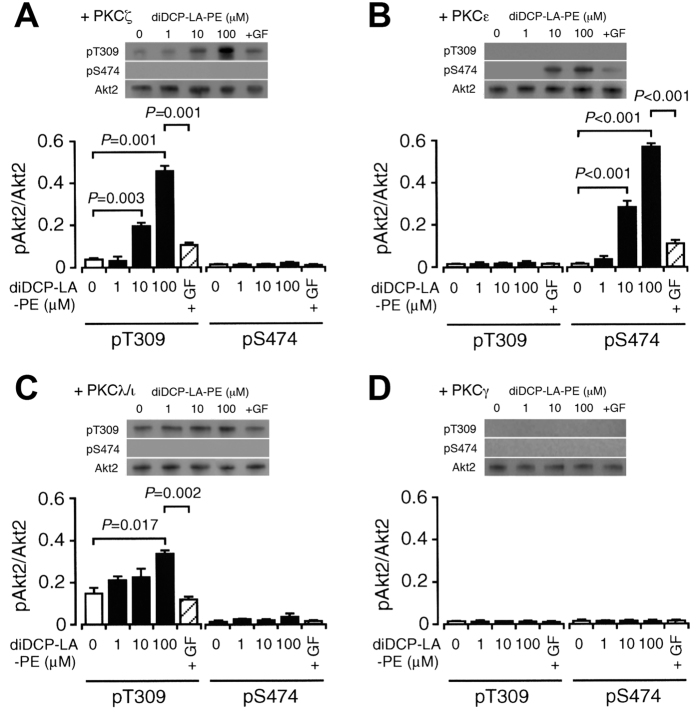
diDCP-LA-PE activates Akt2 directly through PKCζ- and PKCε-mediated phosphorylation. In the cell-free Akt2 assay, Akt2 (1 μg/mL) were reacted with diDCP-LA-PE at concentrations as indicated in the presence of PKCζ (**A**), PKCε (**B**), PKCλ/ι (**C**) or PKCγ (**D**). GF, 100 nM. In the graphs, each column represents the mean (±SEM) signal intensity for phosphorylation at Thr309 or Ser474 relative to that for Akt2 (n = 4 independent experiments). *P* values, ANOVA followed by a Bonferroni correction.

To obtain evidence for interaction of PKCζ and PKCε with Akt, we performed cell-free kinase assay. diDCP-LA-PE phosphorylated Akt2 at Thr309 in the presence of PKCζ in a concentration (1–100 μM)-dependent manner (Fig. 3A). diDCP-LA-PE, alternatively, phosphorylated Akt2 at Ser474 in the presence of PKCε in a concentration (1–100 μM)-dependent manner (Fig. 3B). Like in the presence of PKCζ diDCP-LA-PE phosphorylated Akt2 at Thr309 in the presence of PKCλ/ι (Fig. 3C). In contrast, no phosphorylation was induced in the presence of PKCγ (Fig. 3D). Overall, these results indicate that diDCP-LA-PE is capable of directly activating Akt2 by cooperation of PKCζ (or PKCλ/ι) and PKCε, regardless of a pathway along an IR/IRS-1/PI3K/PDK1/Akt axis.

### diDCP-LA-PE increases cell surface localization of GLUT4 in an insulin-independent manner

**Figure 4:**
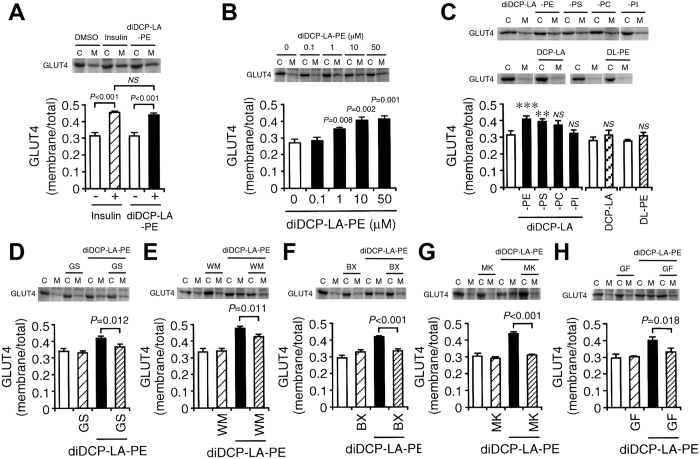
diDCP-LA-PE promotes GLUT4 translocation towards the cell surface in a PI3K-, PDK1-, Akt1/2- and PKC-dependent manner. Differentiated 3T3-L1-GLUT4myc adipocytes were treated with drugs as indicated for 20 min. Then, cells were separated into the cytosolic and plasma membrane fractions, followed by Western blotting. (**A**) Insulin (100 nM) and diDCP-LA-PE (1 μM). (**B**) diDCP-LA-PE at concentrations as indicated. (**C**) diDCP-LA-PS, diDCP-LA-PC, diDCP-LA-PI, DCP-LA and DL-PE at a concentration of 1 μM. diDCP-LA-PE (1 μM) in the presence of GS (50 μM) (**D**), WM (1 μM) (**E**), BX (100 nM) (**F**), MK (5 μM) (**G**) or GF (100 nM) (**H**). In the graphs, each column represents the mean (±SEM) signal intensity for GLUT4 on the plasma membrane relative to that for whole cells (n = 4 independent experiments). *P* values, ANOVA followed by a Bonferroni correction.

If diDCP-LA-PE activates Akt, then the drug should stimulate GLUT4 translocation towards the cell surface. To address this point, we next examined the effect of diDCP-LA-PE on GLUT4 trafficking. Like insulin diDCP-LA-PE increased cell surface localization of GLUT4 in a concentration (0.1–50 μM)-dependent manner in differentiated 3T3-L1 adipocytes (Fig. 4A,B). diDCP-LA-PS increased cell surface localization of GLUT4 to a lesser extent, but no effect was obtained with diDCP-LA-PC, diDCP-LA-PI, DCP-LA or 1,2-dilinoleoyl-*sn*-glycero-3-phosphoethanolamine (DL-PE) (Fig. 4C). This indicates that of the investigated lipids diDCP-LA-PE has the highest potential to translocate GLUT4 towards the cell surface.

diDCP-LA-PE-induced increase in the cell surface localization of GLUT4 was inhibited by the tyrosine kinase inhibitor genistein (GS), the PI3K inhibitor wortmannin (WM), the PDK1 inhibitor BX912 (BX), the Akt inhibitor MK2206 (MK) or the PKC inhibitor GF109203X (GF) (Fig. 4D–H). This suggests that diDCP-LA-PE stimulates GLUT4 translocation towards the cell surface by enhancing a pathway along an IR/IRS-1/PI3K/PDK1/Akt axis or by activating PKC.

**Figure 5:**
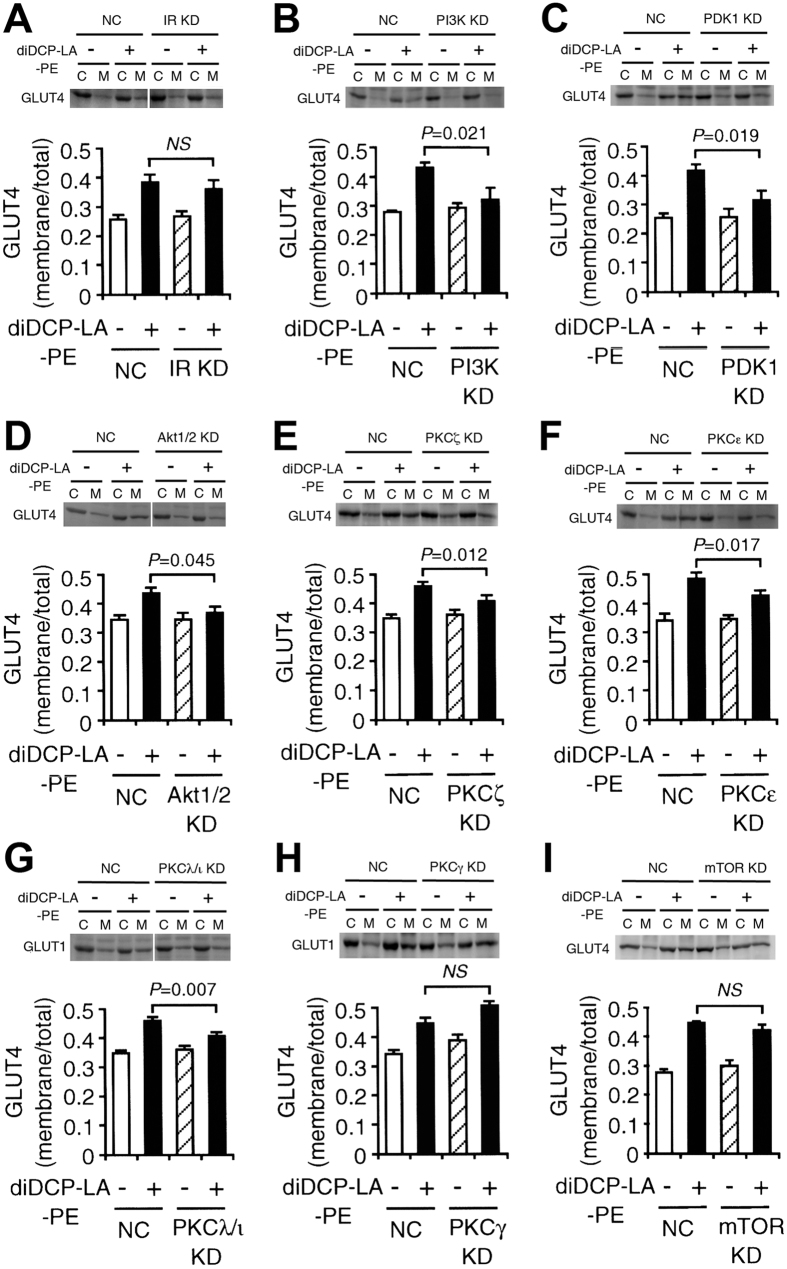
PI3K, PDK1, Akt1/2, PKCζ and PKCε are implicated in the regulation of diDCP-LA-PE-induced GLUT4 translocation. Differentiated 3T3-L1-GLUT4myc adipocytes, which were transfected with siRNAs for IR (**A**), PI3K (**B**), PDK1 (**C**), Akt1/2 (**D**), PKCζ (**E**), PKCε (**F**), PKCλ/ι (**G**), PKCγ (**H**) or mTOR (**I**), were treated with diDCP-LA-PE (1 μM) for 20 min. In the graphs, each column represents the mean (±SEM) signal intensity for GLUT4 on the plasma membrane relative to that for whole cells (n = 4–6 independent experiments). *P* values, ANOVA followed by a Bonferroni correction.

Intriguingly, diDCP-LA-PE-induced increase in the cell surface localization of GLUT4 was not affected by knocking-down IR (Fig. 5A). This implies that diDCP-LA-PE could translocate GLUT4 towards the cell surface, even though IR is not activated or lacking. The effect of diDCP-LA-PE was inhibited by knocking-down PI3K, PDK1 or Akt1/2 (Fig. 5B–D), indicating the implication of PI3K, PDK1 or Akt1/2 in diDCP-LA-PE-induced GLUT4 translocation. Notably, diDCP-LA-PE-induced GLUT4 translocation was prevented by knocking-down PKCζ or PKCε (Fig. 5E,F). This suggests that diDCP-LA-PE promotes GLUT4 translocation still in a PKCζ- or PKCε-dependent manner. The effect of diDCP-LA-PE was also suppressed by knocking-down PKCλ/ι (Fig. 5G), indicating the implication of PKCλ/ι in diDCP-LA-PE-induced GLUT4 translocation. No effect, on the other hand, was obtained by knocking-down PKCγ or mTOR (Fig. 5H,I).

### diDCP-LA-PE stimulates glucose uptake into differentiated 3T3-L1 adipocytes and reduces serum glucose levels in type 1 DM model mice

**Figure 6:**
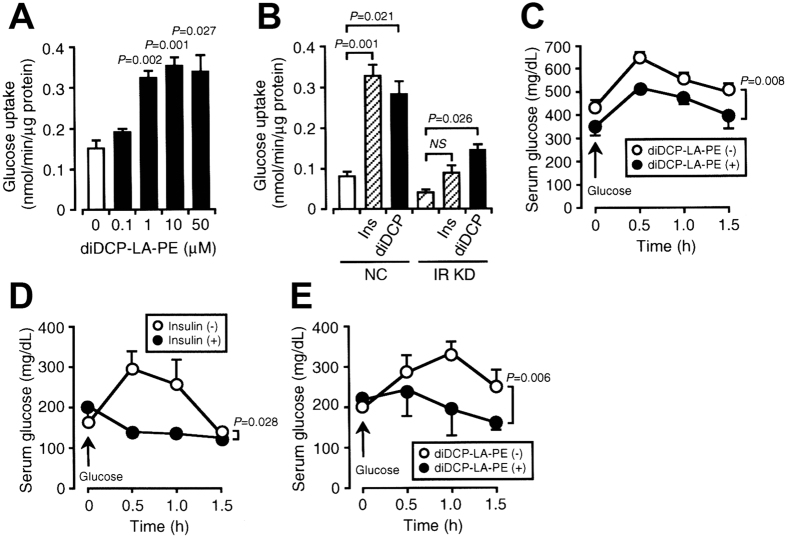
diDCP-LA-PE promotes insulin-independent glucose uptake into differentiated 3T3-L1 adipocytes and reduces serum glucose levels in type 1 and type 2 DM model mice. Differentiated 3T3-L1-GLUT4myc adipocytes without and with IR KD were incubated in a glucose (10 mM)-containing extracellular solution for 1 h, followed by in the presence of diDCP-LA-PE or insulin (Ins) for 2 h. (**A**) diDCP-LA-PE at concentrations as indicated. (**B**) diDCP-LA-PE (1 μM) and Ins (0.1 nM). In the graphs, each column represents the mean (±SEM) glucose uptake (nmol/μg protein/min) (n = 4–6 independent experiments). *P* values, ANOVA followed by a Bonferroni correction. (**C**) OGTT in type 1 DM model mice. diDCP-LA (1 mg/kg body weight) or saline was orally administered 30 min prior to loading glucose (2 g/ml/kg body weight). In the graph, each point represents the mean (±SEM) serum glucose concentration at periods of time as indicated (mg/dL) (n = 5–8 independent mice). *P* value, ANOVA followed by Fisher’s PLSD test. (**D**,**E**) OGTT in type 2 DM model mice. Insulin (0.75 U/kg body weight) (**D**) was intraperitoneally injected into or diDCP-LA-PE (1mg/kg body weight) (**E**) was orally administered to C57BL/KsJ-leprdb/leprdb mice 30 min prior to loading glucose (2 g/ml/kg body weight). In the graphs, each column or each point represents the mean (±SEM) serum glucose concentration (mg/dL) (n = 5 independent mice). *P* values, ANOVA followed by Fisher’s PLSD test.

We finally examined the effect of diDCP-LA-PE on glucose uptake into differentiated 3T3-L1 adipocytes and serum glucose levels in type 1 DM model mice. diDCP-LA-PE stimulated glucose uptake into differentiated 3T3-L1 adipocytes in a concentration (0.1–50 μM)-dependent manner (Fig. 6A). diDCP-LA-PE significantly promoted glucose uptake into cells still with IR knock-down, while no significant effect was obtained with insulin (Fig. 6B). This implies that diDCP-LA-PE could stimulate glucose uptake in an insulin/IR-independent manner.

In the OGTT using type 1 DM model mice, oral administration with diDCP-LA-PE significantly reduced serum glucose levels as compared with that for saline-administered control mice (Fig. 6C).

In the OGTT using type 2 DM model mice, intraperitoneal injection with insulin effectively reduced serum glucose levels (Fig. 6D). Likewise, oral administration with diDCP-LA-PE also reduced serum glucose levels to an extent similar to that for insulin (Fig. 6E).

## Discussion

diDCP-LA-PE serves as a potent inhibitor of PTP1B and direct activator of PKCλ/ι, -ζ, and -ε^2^. diDCP-LA-PE actually enhanced tyrosine phosphorylation of IR and IRS-1 in differentiated 3T3-L1 adipocytes. diDCP-LA-PE enhanced phosphorylation of Akt1/2 at Thr308/309 and Ser473/474, suggesting that diDCP-LA-PE activates Akt1/2 by enhancing an IR/IRS-1/PI3K/PDK/Akt signaling pathway. diDCP-LA-PE-induced Akt1/2 phosphorylation, however, was not significantly inhibited by knocking-down PI3K or PDK1. This interprets that diDCP-LA-PE could also activate Akt1/2 through a pathway independent of an IR/IRS-1/PI3K/PDK/Akt axis. In the cell-free kinase assay, PKCζ and PKCε, which are activated by diDCP-LA-PE, activated Akt2 by phosphorylating at Thr309 and Ser474, respectively. This confirms that PKCζ and -ε engage Akt1/2 activation. diDCP-LA-PE-induced phosphorylation of Akt1/2 at Thr308/309 was cancelled by knocking-down PKCζ in differentiated 3T3-L1 adipocytes, supporting the notion. Strangely, diDCP-LA-PE-induced phosphorylation of Akt1/2 both at Thr308/309 and Ser473/474 was significantly inhibited by knocking-down PKCε. It is presently unknown how PKCε phosphorylates Akt1/2 at Thr308/309 in differentiated 3T3-L1 adipocytes. A plausible explanation is that PKCε might directly interact with PKCζ. To address this question, we are currently carrying out further experiments.

**Figure 7:**
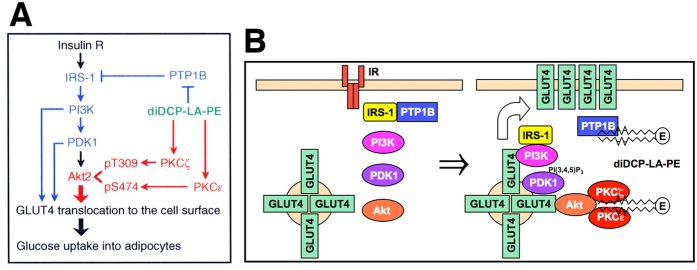
Proposed pathways underlying diDCP-LA-PE-induced GLUT4 translocation and glucose uptake. (**A**) Four pathways include: i) diDCP-LA-PE-induced suppression of PTP1B → relative activation of IRS-1 due to inhibition of tyrosine dephosphorylation → activation of PI3K → stimulation of GLUT4 translocation, ii) diDCP-LA-PE-induced suppression of PTP1B → relative activation of IRS-1 due to inhibition of tyrosine dephosphorylation → activation of PI3K → activation of PDK1 → stimulation of GLUT4 translocation, iii) diDCP-LA-PE-induced suppression of PTP1B → relative activation of IRS-1 due to inhibition of tyrosine dephosphorylation → activation of PI3K → activation of PDK1 → activation of Akt2 → stimulation of GLUT4 translocation, and iv) diDCP-LA-PE-induced co-activation of PKCζ and PKCε → Activation of Akt2 → stimulation of GLUT4 translocation. (**B**) diDCP-LA-PE inhibits PTP1B and activates PKCζ and PKCε through its direct interaction.

Like insulin diDCP-LA-PE promoted translocation of GLUT4 towards the cell surface in differentiated 3T3-L1 adipocytes in a concentration-dependent manner. Such effect was not found with DL-PE. This implies that diDCP-LA-PE exhibits stable bioactivities in cells. diDCP-LA-PE-induced GLUT4 translocation was inhibited by an inhibitor of tyrosine kinase, PI3K, PDK1, or Akt and knocking-down PI3K, PDK1, or Akt1/2. This indicates that diDCP-LA-PE stimulates GLUT4 translocation towards the cell surface through a well-recognized IRS-1/PI3K/PDK1/Akt pathway (Fig. 7A). Moreover, this, in the light of the finding that diDCP-LA-PE-induced Akt1/2 activation in differentiated 3T3-L1 adipocytes was not significantly inhibited by knocking-down PI3K or PDK1, raises the possibility that PI3K or PDK1 each by itself directly regulates GLUT4 translocation in an Akt-independent manner (Fig. 7).

diDCP-LA-PE-induced GLUT4 translocation, alternatively, was inhibited by a PKC inhibitor and knocking-down PKCζ or -ε. This, in the light of the fact that diDCP-LA-PE-induced phosphorylation of Akt1/2 at Thr308/309 and Ser473/474 in differentiated 3T3-L1 adipocytes was suppressed by knocking-down PKCζ and/or PKCε, indicates that diDCP-LA-PE promotes GLUT4 translocation towards the cell surface through PKCζ/-ε-mediated direct action of Akt1/2 (Fig. 7A). diDCP-LA-PE-induced GLUT4 translocation was inhibited still by knocking-down PKCλ/ι, although phosphorylation of Akt1/2 at Thr308/309 was not significantly inhibited by knocking-down PKCλ/ι. This suggests that PKCλ/ι is also participates in the regulation of GLUT4 translocation, regardless of Akt1/2 activation. In contrast, PKCγ did not affect diDCP-LA-PE-induced Akt1/2 activation and GLUT4 translocation. This implies that the effects of PKCζ, -λ/ι, or -ε on GLUT4 translocation are not due to non-specific actions of PKC.

Of particular interest is the finding that diDCP-LA-PE-induced Akt1/2 activation and GLUT4 translocation in differentiated 3T3-L1 adipocytes were not affected by knocking-down IR. Moreover, diDCP-LA-PE significantly increased glucose uptake into differentiated 3T3-L1 adipocytes with IR knock-down. Collectively, these findings indicate that diDCP-LA-PE is capable of mimicking intracellular insulin signaling, i.e., diDCP-LA-PE has the potential to promote GLUT4 translocation towards the cell surface and stimulate glucose uptake still into cells lacking IR or in the absence of insulin. In the OGTT, oral administration with diDCP-LA-PE significantly reduced serum glucose levels in type 1 DM model mice. This raises the possibility that diDCP-LA-PE could control serum glucose levels in type 1 DM patients without insulin injection. Type 1 DM is caused by little/no insulin production in pancreas β cells, and insulin injection is indispensable for type 1 DM therapy. The patients, therefore, suffer physical and mental pain everyday, which would last till the end of their lives. We have been challenging a new therapy for type 1 DM without insulin injection. Consequently, we have devised diDCP-LA-PE, that must become a promising drug for type 1 DM and provide a new hope to relieve the distress for the patients.

In summary, the results of the present study demonstrate that diDCP-LA-PE promotes GLUT4 translocation towards the cell surface and stimulates glucose uptake into differentiated 3T3-L1 adipocytes through PKCζ/-ε-cooperated direct Akt2 activation and in part through PTP1B inhibition-associated activation of a PI3K/PDK1/Akt pathway (Fig. 7B) and that diDCP-LA-PE facilitates glucose uptake still into differentiated 3T3-L1 adipocytes lacking IR or in the absence of insulin and reduces serum glucose levels in type 1 DM model mice. Insulin signal mimetic diDCP-LA-PE, thus, may shed bright light on type 1 DM therapy without insulin injection.

## Additional Information

**How to cite this article**: Nishizaki, T. *et al*. The phosphatidylethanolamine derivative diDCP-LA-PE mimics intracellular insulin signaling. *Sci. Rep.* **6**, 27267; doi: 10.1038/srep27267 (2016).
